# The Daiokanzoto (TJ-84) Kampo Formulation Reduces Virulence Factor Gene Expression in *Porphyromonas gingivalis* and Possesses Anti-Inflammatory and Anti-Protease Activities

**DOI:** 10.1371/journal.pone.0148860

**Published:** 2016-02-09

**Authors:** Jade Fournier-Larente, Jabrane Azelmat, Masami Yoshioka, Daisuke Hinode, Daniel Grenier

**Affiliations:** 1 Groupe de Recherche en Écologie Buccale (GREB), Faculté de Médecine Dentaire, Université Laval, Quebec City, QC, Canada; 2 Department of Oral Health Science and Social Welfare, Institute of Biomedical Sciences, Tokushima University Graduate School, Tokushima, Japan; 3 Department of Hygiene and Oral Health Science, Institute of Biomedical Sciences, Tokushima University Graduate School, Tokushima, Japan; University of Leuven, Rega Institute, BELGIUM

## Abstract

Kampo formulations used in Japan to treat a wide variety of diseases and to promote health are composed of mixtures of crude extracts from the roots, bark, leaves, and rhizomes of a number of herbs. The present study was aimed at identifying the beneficial biological properties of Daiokanzoto (TJ-84), a Kampo formulation composed of crude extracts of Rhubarb rhizomes and Glycyrrhiza roots, with a view to using it as a potential treatment for periodontal disease. Daiokanzoto dose-dependently inhibited the expression of major *Porphyromonas gingivalis* virulence factors involved in host colonization and tissue destruction. More specifically, Daiokanzoto reduced the expression of the *fimA*, *hagA*, *rgpA*, and *rgpB* genes, as determined by quantitative real-time PCR. The U937-3xκB-LUC monocyte cell line transfected with a luciferase reporter gene was used to evaluate the anti-inflammatory properties of Daiokanzoto. Daiokanzoto attenuated the *P*. *gingivalis*-mediated activation of the NF-κB signaling pathway. It also reduced the secretion of pro-inflammatory cytokines (IL-6 and CXCL8) by lipopolysaccharide-stimulated oral epithelial cells and gingival fibroblasts. Lastly, Daiokanzoto, dose-dependently inhibited the catalytic activity of matrix metalloproteinases (-1 and -9). In conclusion, the present study provided evidence that Daiokanzoto shows potential for treating and/or preventing periodontal disease. The ability of this Kampo formulation to act on both bacterial pathogens and the host inflammatory response, the two etiological components of periodontal disease, is of high therapeutic interest.

## Introduction

Traditional Japanese herbal medicine, also called Kampo medicine, is practiced in Japan by licensed medical professionals as well as by authorized Kampo practitioners to treat a wide variety of diseases and to promote health [1, 2]. While only four Kampo formulations had been approved by 1967, there are now 148 different formulations recognized by the Japanese national health insurance system [1, 2]. Kampo formulations, which are composed of mixtures of crude extracts from the roots, bark, leaves, or rhizomes of a number of herbs, are manufactured by Japanese pharmaceutical companies and are strictly controlled by government regulations [1, 2]. Some of the phytochemicals in Kampo formulations likely act in synergy on several therapeutic targets.

Periodontitis is a common inflammatory disorder that affects tooth-supporting tissues and that may lead to tooth loss. Periodontitis has two major etiological components: (i) specific Gram-negative anaerobic bacterial species, called periodontopathogens, that colonize subgingival sites as biofilms [3], and (ii) the continuous host immune response to these periodontopathogens that results in the production of high levels of inflammatory mediators and matrix metalloproteinases (MMPs) that mediate the destruction of connective tissue and alveolar bone [4, 5]. Very few studies of the beneficial effect of Kampo medicines on periodontal disease have been published. Shosaikoto (TJ-9) and Orento (TJ-120) have been shown to suppress lipopolysaccharide (LPS)-induced prostaglandin E_2_ production by human gingival fibroblasts [6, 7], while Juzentaihoto (TJ-48) inhibits osteoclast differentiation *in vitro* and reduces alveolar bone loss in a rat periodontitis model [8]. In a recent study, we showed that Rokumigan (TJ-87) promotes wound healing in a gingival fibroblast model, attenuates pro-inflammatory cytokine secretion by oral mucosal cells, and prevents biofilm formation by *Fusobacterium nucleatum* [9], a bacterial species that plays an important role in bridging commensal Gram-positive “early colonizers” and the periodontopathogenic Gram-negative “late colonizers” in subgingival biofilm [10]. Lastly, we reported that Daiokanzoto (TJ-84) inhibits the growth and adherence properties of *Porphyromonas gingivalis* [11], a key etiologic agent of chronic periodontitis. More specifically, this bacterial expresses a broad array of virulence factors that allow it to colonize subgingival sites, circumvent the immune system, and cause tissue destruction [12]. Moreover, evidence has been brought that *P*. *gingivalis* can subvert the host immune response to remodel a normally symbiotic bacterial community into a dysbiotic microflora [13].

In this continuation of our ongoing research aimed at identifying the beneficial properties of Daiokanzoto with a view to using it as a potential treatment for periodontal disease, we investigated the impact of this Kampo formulation on virulence factor gene expression by *P*. *gingivalis*, activation of the nuclear factor kappa B (NF-κB) signaling pathway and secretion of pro-inflammatory cytokines by host cells, and activity of tissue degrading enzyme MMP-1 and MMP-9.

## Materials and Methods

### Daiokanzoto (TJ-84)

Daiokanzoto was obtained from Tsumura Co. Ltd. (# D26692; Tokyo, Japan) as packaged pellets. This Kampo is produced by combining crude extracts of Rhubarb rhizomes and Glycyrrhiza roots. A Daiokanzoto stock solution was prepared by adding 50 mg of pellets to 5 ml of hot distilled water (80°C). The mixture was stirred for 1 h at 37°C and was then filter-sterilized through a 0.22-μm pore size membrane. The filtrate was stored in the dark at 4°C for up to one week.

### Bacteria and growth conditions

The *P*. *gingivalis* ATCC 33277 reference strain was routinely grown in Todd-Hewitt broth (Becton Dickinson, Mississauga, ON, Canada) supplemented with 0.001% hemin and 0.0001% vitamin K (THB-HK) at 37°C in an anaerobic chamber (N_2_:H_2_:CO_2_ / 75:10:15).

### Virulence factor gene expression by *P*. *gingivalis*

The effect of Daiokanzoto on the expression of several *P*. *gingivalis* virulence factor genes involved in host colonization (adhesins *fimA* and *hagA*) and tissue destruction (proteases *rgpA* and *rgpB*) was investigated by reverse transcription (RT)-quantitative real-time polymerase chain reaction (qPCR). The bacteria were grown to the mid-log phase (OD_660_ = 0.45). The Daiokanzoto was added (100, 400, or 800 μg/ml final concentration), and the bacteria were incubated at 37°C under anaerobic conditions for an additional 8 h. These concentrations were below the minimal inhibitory concentration (MIC), which was evaluated at 1,000 μg/ml in a previous study (11). Control cells were incubated in the absence of Daiokanzoto. The bacteria were collected by centrifugation (7,000 x *g* for 5 min) and were treated with the RNAprotect bacterial reagent (Qiagen Canada Inc., Montreal, QC, Canada). The bacterial cells were then lysed, and their RNA was isolated and purified using RNeasy minikits (Qiagen Canada Inc.). The purity of mRNA was evaluated using the Experion^TM^ system (Bio-Rad Laboratories, Mississauga, ON, Canada). Total cDNA was prepared from 1 μg of mRNA by RT-PCR using iScript^TM^ Reverse Transcription Supermix (Bio-Rad Laboratories) according to the manufacturer’s instructions. The RT-PCR conditions were as follows: 5 min at 25°C, 30 min at 42°C, and 5 min at 85°C. qPCR was used to quantify the levels of *fimA*, *hagA*, *rgpA*, and *rgpB* genes. The 16S rRNA gene was used as a reference gene to normalize the results. The primers used for the qPCR (Life Technologies Inc., Burlington, ON, Canada) have been described in a previous study [14]. The PCR mixtures contained 5 μl of IQ SYBR Green Supermix (Bio-Rad Laboratories), 4 μl of cDNA, 0.4 μl of gene-specific primer (10 mM), and 0.6 μl of RNase- and DNase-free water. The samples were amplified using a Bio-Rad MyCycler^TM^ thermal cycler (Bio-Rad Laboratories). The amplification conditions were as follows: 95°C for 5 min followed by 35 cycles at 95°C for 1 min, 52°C for 1 min, and 72°C for 30 s. Temperature curve analyses were performed to validate the specificity of each primer pair. Assays were performed in triplicate for a minimum of three independent experiments to ensure reproducibility. A representative set of data is presented.

### Activation of the NF-κB signaling pathway in monocytes

The human monoblastic leukemia cell line U937-3xκB-LUC, a subclone of the U937 cell line stably transfected with a construct containing three NF-κB binding sites from the Ig κ light chain promoter coupled with the firefly luciferase gene (3x-κB-*luc*), was kindly provided by R. Blomhoff (University of Oslo, Norway) [15]. The cells were routinely grown at 37°C in a 5% CO_2_ atmosphere in RPMI-1640 medium (Life Technologies Inc.) supplemented with 10% heat-inactivated fetal bovine serum (FBS), 100 μg/ml of penicillin-streptomycin, and 75 μg/ml of hygromycin B (Sigma-Aldrich Canada Ltd., Oakville, ON, Canada). U937-3xκB-LUC monocytes (2 x 10^6^ cells/ml) were suspended in the supplemented RPMI-1640 culture medium and were seeded (100 μl) in the wells of black-bottom, black-walled, 96-well microplates. Two-fold serial dilutions of Daiokanzoto were then added to the wells (500 to 15.625 μg/ml). A preliminary analysis showed that 500 μg/ml is the highest non-cytotoxic concentration of Daiokanzoto that can be used with this cell line (data not shown). An assay was also performed using a commercial inhibitor (25 μM, BAY-11-7082; EMD Millipore, Billerica, MA, USA) of the NF-κB signaling pathway. Following a 30-min incubation, *P*. *gingivalis* cells suspended in the supplemented RPMI-1640 medium were added to the wells at an MOI of 100 to induce the activation of the NF-κB signaling pathway. The plate was incubated at 37°C (5% CO_2_) for a further 6 h. Luciferase substrate (100 μl) was added to the wells, and NF-κB activation was monitored at room temperature using the Bright-Glo™ Luciferase Assay System (Promega, Madison WI, USA). Luminescence was recorded using the luminometer option of a Synergy 2 microplate reader (BioTek Instruments, Winooski, VT, USA) within 3 min of adding the substrate.

### Production of pro-inflammatory cytokines by lipopolysaccharide-stimulated oral epithelial cells and gingival fibroblasts

The immortalized human oral epithelial cell line OBA-9 [16], which was kindly provided by M. Mayer (University of São Paulo, Brazil), was cultured in keratinocyte serum-free medium (K-SFM; Life Technologies Inc.) supplemented with growth factors (insulin, epidermal growth factor, fibroblast growth factor) and 100 μg/ml of penicillin G-streptomycin. The HGF-1 primary human gingival fibroblast cell line (CRL-2014; American Type Culture Collection, Manassas, VA, USA) was cultured in Dulbecco’s modified Eagle’s medium (DMEM) supplemented with 4 mM L-glutamine (HyClone Laboratories, Logan, UT, USA), 10% heat-inactivated FBS, and 100 μg/ml of penicillin G-streptomycin. Both cell lines were incubated at 37°C in a 5% CO_2_ atmosphere until they reached confluence. The cells were seeded in the wells of a 12-well microplate (1 ml/well, 1 x 10^6^ cells/ml) and were incubated overnight at 37°C in a 5% CO_2_ atmosphere to allow cell adhesion. Thereafter, the cells were pre-treated for 2 h with two-fold serial dilutions of Daiokanzoto (100 to 25 μg/ml) prior to being stimulated with *P*. *gingivalis* lipopolysaccharide (LPS, 1 μg/ml) for 24 h at 37°C in a 5% CO_2_ atmosphere. A preliminary analysis showed that 100 μg/ml was the highest non-cytotoxic concentration of Daiokanzoto for the two cell lines (data not shown). The supernatants were collected, centrifuged (500 x g for 5 min at 4°C), and stored at –20°C until used to quantify interleukin-6 (IL-6) and interleukin-8 (CXCL8) using commercial enzyme-linked immunosorbent assay (ELISA) kits (eBioscence Inc., San Diego, CA, USA) according to the manufacturer’s instructions.

### Determination of MMP activity

Human recombinant MMP-9 (gelatinase B; Calbiochem, San Diego, CA, USA) was obtained in the active form while human recombinant MMP-1 (collagenase; Calbiochem) was activated at 37°C for 2 h in 10-fold TCNB buffer (50 mM Tris·HCl, 10 mM CaCl_2_, 150 mM NaCl and 0.05% Brij35, pH 7.5) containing 1 mM p-aminophenylmercuric acetate. Active MMPs were diluted to a final concentration of 100 ng/ml in TCNB buffer and increasing concentrations of Daiokanzoto (0, 125, 250, and 500 μg/ml). Fluorogenic substrates (100 μg/ml; fluorescein-conjugated DQ^TM^ type I collagen and fluorescein-conjugated DQ^TM^ gelatin; Molecular Probes, Eugene, OR, USA) were then added. The assay mixtures were incubated in the dark for 4 h at room temperature for MMP-1 and 37°C for MMP-9. The fluorescence was measured using the fluorometer option of a Synergy 2 microplate reader (BioTek Instruments) with the excitation and emission wavelengths set at 490 nm and 520 nm, respectively.

### Statistical analysis

Unless indicated otherwise, assays were performed in triplicate, and the means ± standard deviations were calculated. Differences between the means were analyzed for statistical significance using the Student’s t-test and were considered significant at *p* < 0.01.

## Results

**Fig 1:**
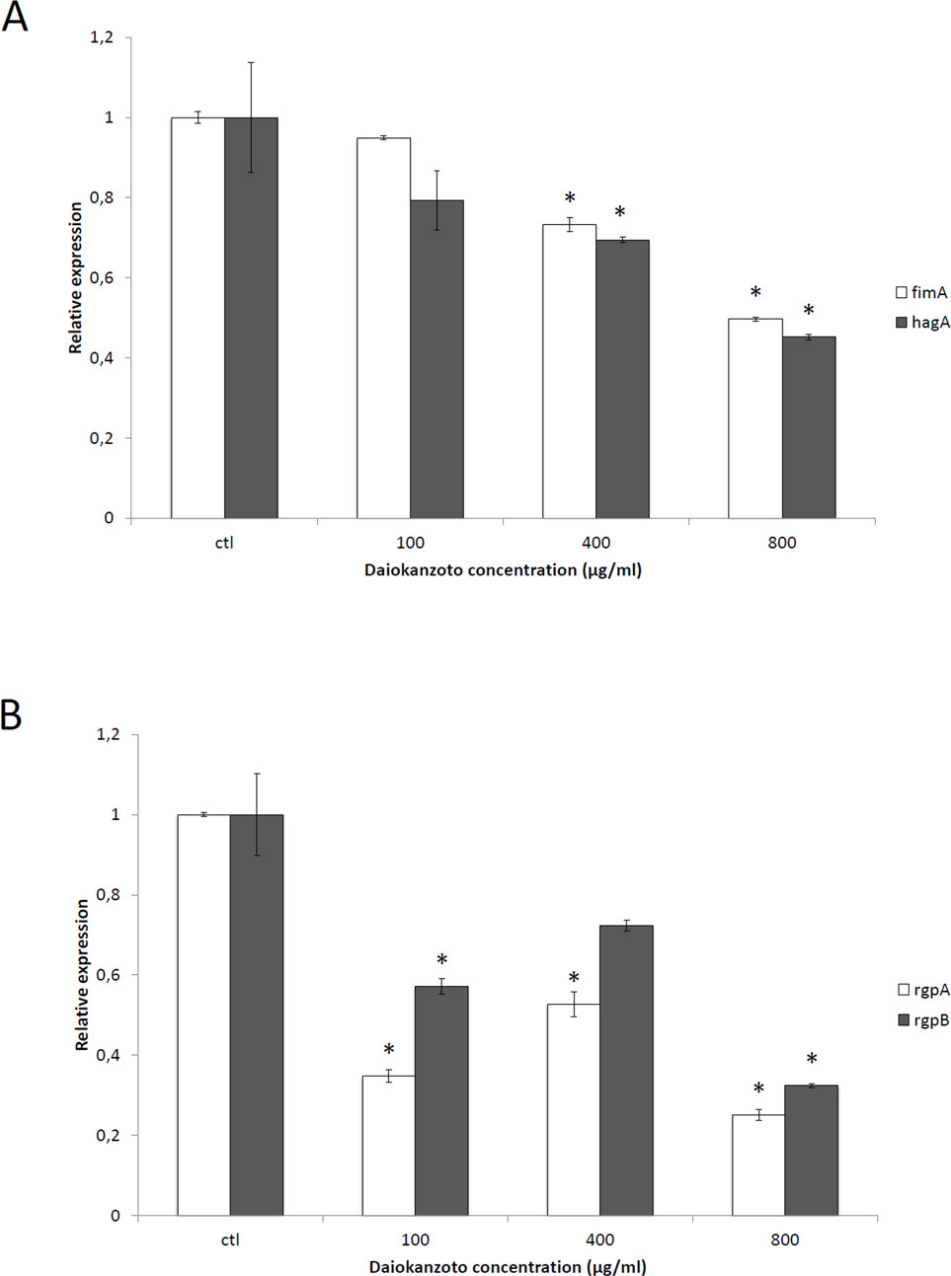
Effect of Daiokanzoto on the expression of *fimA* and *hagA* (Panel A) and *rgpA* and *rgpB* mRNA (Panel B) in *P*. *gingivalis*. Data are expressed as means ± standard deviations. mRNA expression was normalized to 16S rRNA. *, significantly different (P < 0.01) compared to an untreated control.

We first investigated the effect of Daiokanzoto on the expression of several virulence factor genes by *P*. *gingivalis*. An early exponential growth phase culture of *P*. *gingivalis* was exposed to Daiokanzoto at sub-MICs (100, 400 or 800 μg/ml) for 8 h under anaerobic conditions prior to monitoring the expression of mRNA by RT-qPCR. Fig 1A shows the effects on two genes (*fimA*, *hagA*) coding for factors involved in bacterial colonization. A significant dose-dependent decrease in the expression of both genes was observed. More specifically, the expression of *fimA* and *hagA* decreased by 50.3% and 54.8%, respectively, in the presence of 800 μg/ml of Daiokanzoto. The expression of *rgpA* and *rgpB*, two protease genes related to the inactivation of host defense mechanisms, tissue destruction, and nutrient acquisition, also decreased by 74.9% and 67.6%, respectively, in the presence of 800 μg/ml of Daiokanzoto (Fig 1B).

**Fig 2:**
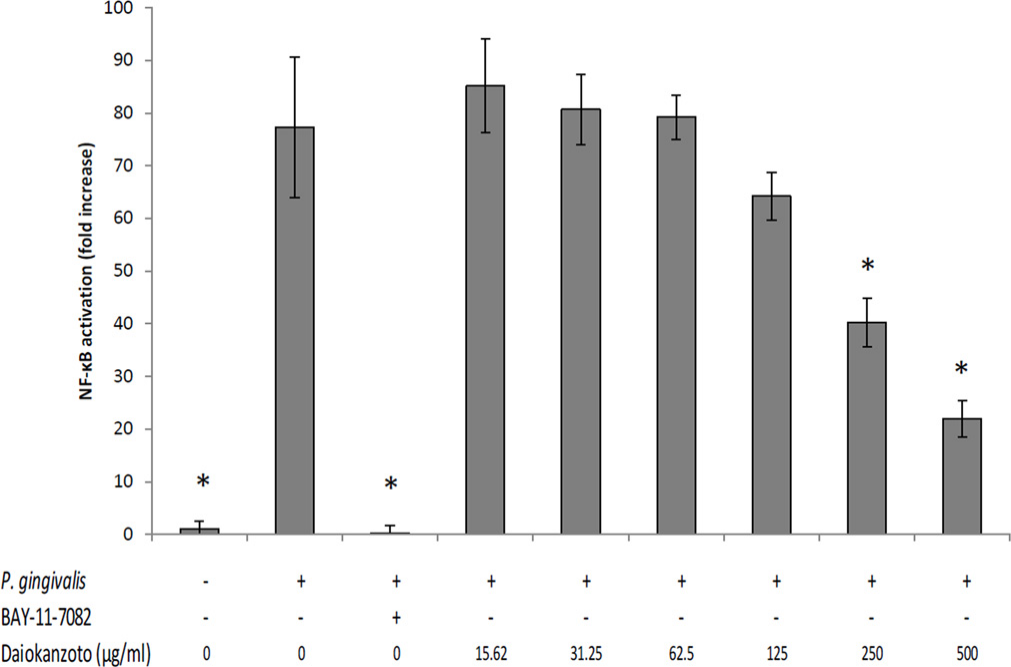
Effect of Daiokanzoto on *P*. *gingivalis*-induced activation of the NF-κB signaling pathway in monocytes. Data are expressed as means ± standard deviations. *, Significant inhibition at *p* < 0.01 using a Student’s *t*-test.

To evaluate the anti-inflammatory properties of Daiokanzoto, the U937-3xκB-LUC cell line transfected with a luciferase reporter gene was used to determine the effect of this Kampo formulation on *P*. *gingivalis*-mediated activation of the NF-κB signaling pathway. Daiokanzoto dose-dependently inhibited the activation of NF-κB induced by *P*. *gingivalis* at an MOI of 100 (Fig 2). The activation of NF-κB was reduced by 59.8% and 78.1%, respectively, in the presence of 250 and 500 μg/ml of Daiokanzoto. The commercial inhibitor BAY-11-7082 (25 μM), which was used as a positive control, completely prevented NF-κB activation.

**Fig 3:**
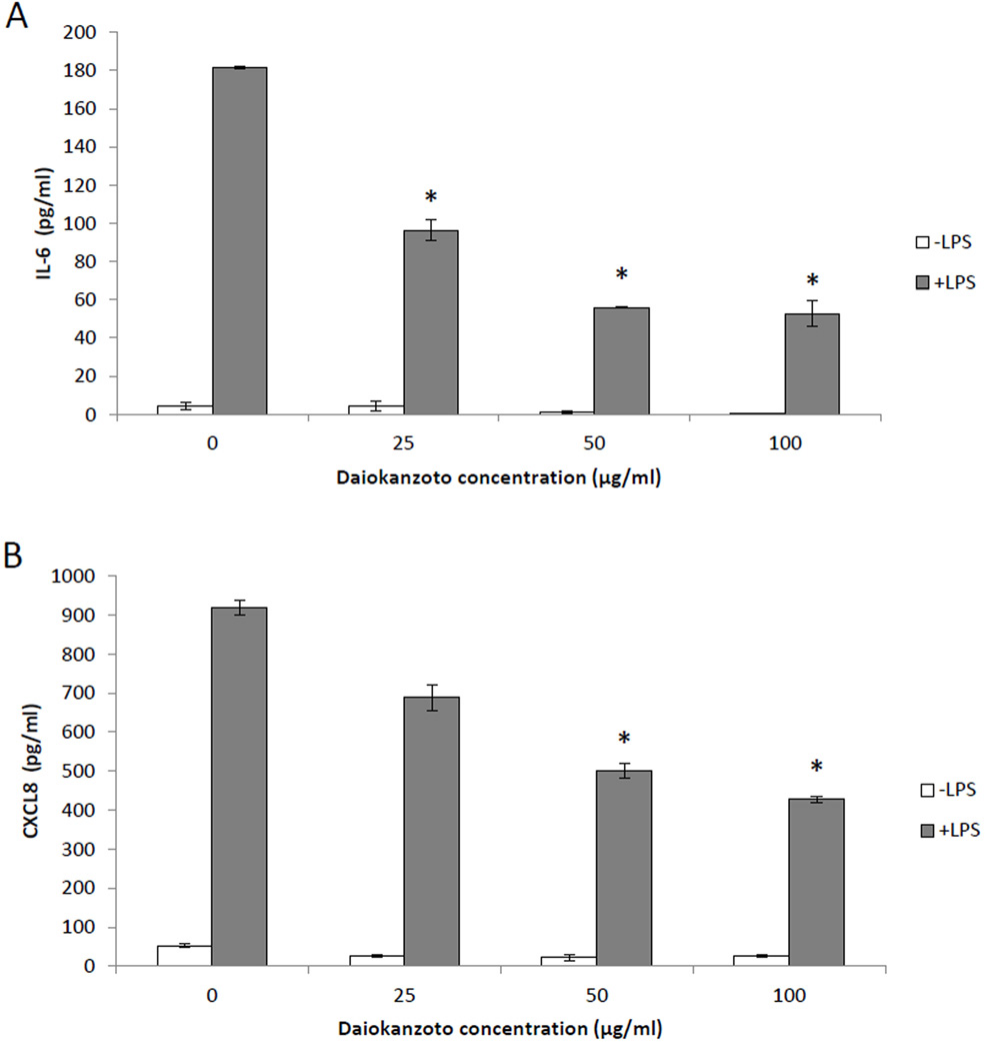
Effect of Daiokanzoto on IL-6 (Panel A) and CXCL8 (Panel B) secretion by LPS-stimulated human oral epithelial cells (OBA-9). Data are expressed as means ± standard deviations. *, Significant inhibition at *p* < 0.01 using a Student’s *t*-test.

**Fig 4:**
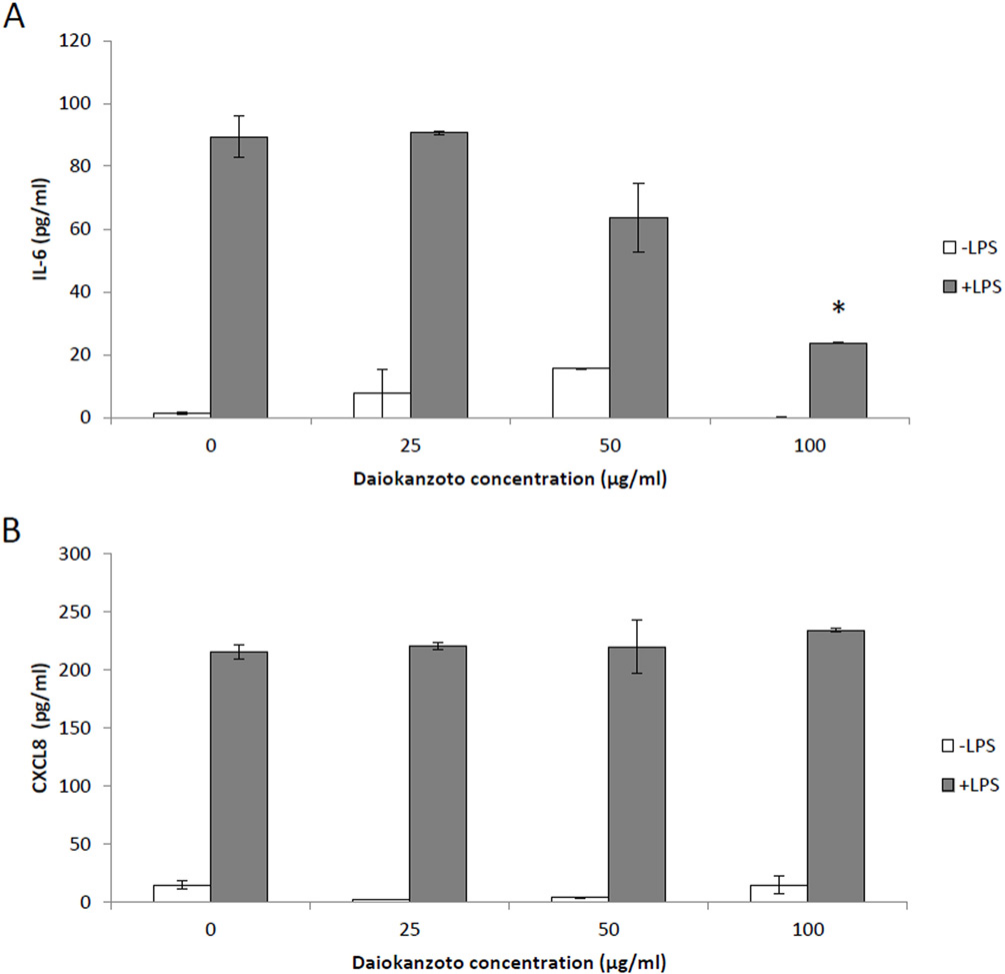
Effect of Daiokanzoto on IL-6 (Panel A) and CXCL8 (Panel B) secretion by LPS-stimulated human gingival fibroblasts (HGF-1). Data are expressed as means ± standard deviations. *, Significant inhibition at *p* < 0.01 using a Student’s *t*-test.

The ability of Daiokanzoto to inhibit IL-6 and CXCL8 secretion by oral epithelial cells and gingival fibroblasts was also tested. LPS stimulation of epithelial cells increased IL-6 secretion 45-fold and CXCL8 secretion 18-fold (Fig 3), while the stimulation of gingival fibroblasts with LPS increased IL-6 secretion 67-fold and CXCL8 secretion 15-fold (Fig 4). Daiokanzoto significantly decreased the secretion of IL-6 by both LPS-stimulated epithelial cells and fibroblasts (Figs 3 and 4). Daiokanzoto (100 μg/ml) decreased IL-6 secretion by fibroblasts and epithelial cells by 73.3% and 71%, respectively. While it had no effect on the secretion of CXCL8 by gingival fibroblasts, Daiokanzoto significantly and dose-dependently decreased the secretion of CXCL8 by epithelial cells (Fig 3). At 100 μg/ml, it inhibited CXCL8 secretion by 53.5%.

Lastly, since tissue destruction associated with periodontitis is mostly mediated by MMPs, we evaluated the effect of Daiokanzoto on the catalytic activity of MMP-1 and MMP-9, which are secreted by several human cell types. Daiokanzoto at 500 μg/ml inhibited type I collagen degradation by MMP-1 by 72% (Table 1). Similarly, Daiokanzoto (500 μg/ml) caused inhibition of MMP-9-mediated gelatin degradation (83.1%) (Table 1).

**Table 1:** Effect of Daiokanzoto on the activity of MMP-1 and MMP-9.

Concentration (μg/ml)	Relative activity (%) ^1^	
	MMP-1	MMP-9
0	100 ± 8.4	100 ± 9.6
125	79.8 ± 17.1	85.3 ± 8.1
250	65.2 ± 10.6 ^2^	54.7 ± 12.4 ^2^
500	28.0 ± 12.9 ^2^	16.9 ± 6.6 ^2^

^1^ Data are expressed as means ± standard deviations.

^2^ Significant inhibition at *p* < 0.01 using a Student’s *t*-test.

## Discussion

Approximately 5–15% of the general population is affected by severe forms of periodontal disease [17] that, if left untreated, may result in tooth loss and systemic complications such as cardiovascular diseases, preterm delivery, rheumatoid arthritis, and respiratory infections [18]. The conventional treatment for periodontitis involves the mechanical removal of the subgingival biofilm and tartar that induce the host immune-destructive response. While this procedure is effective in managing the majority of periodontitis patients, conventional therapy does not always achieve the desired clinical outcome. For instance, controlling periodontitis in high-risk individuals (smokers, diabetics or individuals possessing genetic predisposition) or individuals who do not respond to conventional therapy may require adjunctive treatments [19].

Recent evidence has suggested that Kampo formulations may possess potentially valuable properties for treating oral diseases. More specifically, antibacterial, anti-inflammatory, and wound healing activities have been associated with some Kampo formulations [6–9, 11]. In a recent study, we showed that Daiokanzoto (TJ-84) exerts antibacterial activity against *P*. *gingivalis* and attenuates some the virulence properties of this bacterium [11]. The goal of the present study was to provide additional evidence of the potential of Daiokanzoto for treating periodontal disease.

Given the critical role of *P*. *gingivalis* in chronic periodontitis [12], compounds that attenuate the virulence properties of this bacterium are of great interest. We showed that Daiokanzoto reduces the expression of *fimA* and *hagA*, which are involved in host colonization by *P*. *gingivalis* [20]. Interestingly, in a previous study, Daiokanzoto was shown to reduce the adherence of *P*. *gingivalis* to gingival epithelial cells [11], suggesting that this Kampo formulation could potentially be used for an anti-infective strategy based on preventing the adherence of pathogens to mucosal surfaces. The expression of *rgpA* and *rgpB*, two protease genes that are associated with the inactivation of host defense mechanisms, tissue destruction, and nutrient acquisition [21] was also down-regulated when *P*. *gingivalis* was incubated with Daiokanzoto at sub-MICs. Interestingly, we previously reported the ability of Daiokanzoto to inhibit the gingipain-mediated gelatin degradation by *P*. *gingivalis* [11].

The transcription factor NF-κB is activated by a wide variety of stimuli, including bacterial pathogens, and has many target genes, including genes encoding cytokines, adhesion molecules, and MMPs [22]. Therapeutic approaches that inhibit inflammatory mediator production by host cells represent an interesting alternative for controlling inflammatory diseases such as periodontal diseases [23]. We showed that Daiokanzoto inhibits NF-κB activation by *P*. *gingivalis*. Daiokanzoto is a mixture of crude extracts of Rhubarb rhizomes and Glycyrrhiza roots. Interestingly, some of the active constituents of *Glycyrrhiza* spp., including flavanones, chalcones, isoflavans, flavones, and isoflavones, have been reported to inhibit NF-κB activation in various cell types [24].

Fibroblasts and epithelial cells are major constituents of gingival tissues and act as a physical barrier to prevent invasion by periodontopathogens [25]. In response to stimulation by periodontopathogens and their virulence factors, these cells secrete pro-inflammatory cytokines such as IL-6 and CXCL8 [26, 27]. The periodontal tissues and gingival crevicular fluid of periodontitis patients contain high levels of inflammatory mediators, and their etiological correlation with periodontitis has been clearly demonstrated [28]. It is thus logical to investigate therapeutic approaches that modulate the host inflammatory response in order to manage the progression of chronic periodontitis. In the present study, we showed that Daiokanzoto inhibits LPS-induced IL-6 secretion by both gingival fibroblasts and epithelial cells. The expression of IL-6 has been shown to be higher in periodontal inflammation sites and to be closely related to the clinical severity of periodontitis [29]. In addition, IL-6 levels are also higher in the diseased gingiva of patients with periodontitis than in the gingiva of periodontally healthy subjects [30]. IL-6 is a multifunctional cytokine that can induce osteoclast formation and promote bone resorption [31, 32]. While this occurs naturally as part of normal bone remodeling, excess IL-6 production, as during periodontitis, leads to the destruction of alveolar bone. By attenuating the secretion of IL-6, Daiokanzoto may contribute to reducing bone resorption. Oral epithelial cells are known to secrete chemokines, including CXCL8, which are chemoattractants for polymorphonuclear leukocytes and macrophages. Interestingly, increased levels of CXCL8 are found in the gingival crevicular fluid of inflamed periodontal sites compared with healthy sites [33]. Moreover, periodontal therapy reduces immune cell numbers, and the levels of CXCL8 in gingival crevicular fluid [34]. Given the above, the ability of Daiokanzoto to inhibit CXCL8 secretion by epithelial cells may attenuate periodontal inflammation. Two other Kampo formulations, Shosaikoto (TJ-9) and Orento (TJ-120), have also been reported to possess anti-inflammatory activity through their ability to suppress LPS-induced prostaglandin E_2_ (PGE_2_) production by gingival fibroblasts [6, 7].

MMPs are proteolytic enzymes released by major cell types in the periodontium, including fibroblasts, neutrophils, and macrophages [5]. Since these enzymes can degrade most components of the extracellular matrix (ECM), high levels of MMPs in active periodontal sites lead to the destruction of periodontal tissues by the degradation of periodontal ligaments, the loss of gingival collagen, and the resorption of alveolar bone [5]. High levels of active MMP-9 in gingival crevicular fluid have been associated with periodontal tissue destruction [35]. In addition, periodontitis-affected gingival tissue express high levels of MMP-1 mRNA [36]. We showed that Daiokanzoto inhibits the catalytic activity of both MMP-1 and MMP-9 and may thus contribute to reducing host cell damage, including bone resorption.

Yoshida et al. [37] recently showed that Daiokanzoto can attenuate 5-fluorouracil-induced cell death by inhibiting the production of mitochondrial reactive oxygen species. This led to the suggestion that Daiokanzoto could be used to treat oral mucositis in patients receiving multicycle chemotherapy. The present study provided evidence that Daiokanzoto could potentially be used to treat periodontal disease, another major oral disorder. Clinical trials are now required to assess the potential oral health benefits Daiokanzoto. In this regard, bioactive molecules could be applied locally to diseased periodontal sites by irrigation or by the insertion of resorbable fibers to attenuate the virulence properties of *P*. *gingivalis* as well as the inflammation process.

## References

[1] Yakubo S, Ito M, Ueda Y, Okamoto H, Kimura Y, Amano Y, et al Pattern classification in Kampo medicine. Evid Based Complement Alternat Med 2014; 2014: 535146 10.1155/2014/535146 24701241 PMC3950553

[2] Watanabe K, Matsuura K, Gao P, Hottenbacher L, Tokunaga H, Nishimura K, et al Traditional Japanese kampo medicine: Clinical research between modernity and traditional medicine—The state of research and methodological suggestions for the future. Evid Based Complement Alternat Med 2011; 2011: 513842 10.1093/ecam/neq067 21687585 PMC3114407

[3] Berezow AB, Darveau RP. Microbial shift and periodontitis. Periodontol 2000 2011; 55: 36–47. 10.1111/j.1600-0757.2010.00350.x 21134227 PMC3058494

[4] Liu YC, Lerner UH, Teng YT. Cytokine responses against periodontal infection: protective and destructive roles. Periodontol 2000 2010; 52: 163–206. 10.1111/j.1600-0757.2009.00321.x 20017801

[5] Sapna G, Gokul S, Bagri-Manjrekar K. Matrix metalloproteinases and periodontal diseases. Oral Dis 2014; 20: 538–550. 10.1111/odi.12159 23849049

[6] Ara T, Maeda Y, Fujinami Y, Imamura Y, Hattori T, Wang PL. Preventive effects of a Kampo medicine, Shosaikoto, on inflammatory responses in LPS-treated human fibroblasts. Biol Pharm Bull 2008; 31: 1141–1144. 18520044 10.1248/bpb.31.1141

[7] Ara T, Honjo KI, Fujinami Y, Hattori T, Imamura Y, Wang PL. Preventive effects of a Kampo medicine, Orento on inflammatory responses in lipopolysaccharide-treated human fibroblasts. Biol Pharm Bull 2010; 33: 611–616. 20410594 10.1248/bpb.33.611

[8] Takeda O, Toyama T, Watanabe K, Sato T, Sasaguri K, Akimoto S, et al Ameliorating effects of Juzentaihoto on restraint stress and *P*. *gingivalis*-induced alveolar bone loss. Archs Oral Biol 2014; 59: 1130–1138. 10.1016/j.archoralbio.2014.06.010 25064760

[9] Liao J, Azelmat J, Zhao L, Yoshioka M, Hinode D, Grenier D. The Kampo medicine Rokumigan possesses antibiofilm, anti-inflammatory and wound healing properties. BioMed Res Int 2014: 436206 10.1155/2014/436206 24877093 PMC4022067

[10] Kolenbrander PE. Oral microbial communities: biofilms, interactions, and genetic systems. Annu Rev Microbiol 2000;54: 413–437. 11018133 10.1146/annurev.micro.54.1.413

[11] Liao J, Zhao L, Yoshioka M, Hinode D, Grenier D. Effects of Japanese traditional herbal medicines (Kampo) on growth and virulence properties of *Porphyromonas gingivalis* and viability of oral epithelial cells. Pharm Biol 2013; 51: 1538–1544. 10.3109/13880209.2013.801995 23987742

[12] Bostanci N, Belibasakis GN. *Porphyromonas gingivalis*: an invasive and evasive opportunistic oral pathogen. FEMS Microbiol Lett 2012; 333: 1–9. 10.1111/j.1574-6968.2012.02579.x 22530835

[13] Zenobia C, Hajishengallis G. *Porphyromonas gingivalis* virulence factors involved in subversion of leukocytes and microbial dysbiosis. Virulence 2015; 6: 236–243. 10.1080/21505594.2014.999567 25654623 PMC4601496

[14] Azelmat J, Fournier-Larente J, Grenier D. The anthraquinone rhein attenuates virulence gene expression in *Porphyromonas gingivalis* and exhibits synergistic antibacterial activity in association with metronidazole or natural compounds. Archs Oral Biol 2015; 60: 342–346. 10.1016/j.archoralbio.2014.11.006 25463909

[15] Carlsen H, Moskaug JO, Fromm SH, Blomhoff R. *In vivo* imaging of NF-kappa B activity. J Immunol 2002; 168: 1441–1446. 11801687 10.4049/jimmunol.168.3.1441

[16] Kusumoto Y, Hirano H, Saitoh K, Yamada S, Takedachi M, Nozaki T, et al Human gingival epithelial cells produce chemotactic factors interleukin-8 and monocyte chemoattractant protein-1 after stimulation with *Porphyromonas gingivalis* via toll-like receptor 2. J Periodontol 2004; 75: 370–379. 15088874 10.1902/jop.2004.75.3.370

[17] Eke PI, Dye BA, Wei L, Thornton-Evans GO, Genco RJ. Prevalence of periodontitis in adults in the United States: 2009 and 2010. J Dent Res 2012; 91: 914–920. 22935673 10.1177/0022034512457373

[18] Pizzo G, Guiglia R, Russo LL, Campisi G. Dentistry and internal medicine: From the focal infection theory to the periodontal medicine concept. Eur J Intern Med 2010; 21: 496–502. 10.1016/j.ejim.2010.07.011 21111933

[19] Herrera D, Alonso B, Leon R, Roldan S, Sanz M. Antimicrobial therapy in periodontitis: the use of antimicrobials against the subgingival biofilm. J Clin Periodontol 2008; 35: 45–66. 10.1111/j.1600-051X.2008.01260.x 18724841

[20] Lamont RJ, Jenkinson HF. Life below the gum line: Pathogenic mechanisms of *Porphyromonas gingivalis*. Microbiol Mol Biol Rev 1998; 62: 1244–1263. 9841671 10.1128/mmbr.62.4.1244-1263.1998 PMC98945

[21] Imamura T. The role of gingipains in the pathogenesis of periodontal disease. J Periodontol 2003; 74: 111–118. 12593605 10.1902/jop.2003.74.1.111

[22] Kumar A, Takada Y, Boriek AM, Aggarwal BB. Nuclear factor-kappaB: its role in health and disease. J Mol Med 2004; 82, 434–448. 15175863 10.1007/s00109-004-0555-y

[23] Souza JA, Rossa C, Garlet GP, Nogueira AV, Cirelli JA. Modulation of host cell signaling pathways as a therapeutic approach in periodontal disease. J Appl Oral Sci 2012; 20: 128–138. 22666826 10.1590/S1678-77572012000200002 PMC3894752

[24] Hosseinzadeh H, Nassiri-Asl M. Pharmacological effects of *Glycyrrhiza* spp. and ist bioactive constituents: Update and review. Phytother Res 2015; 10.1002/ptr.5487 26462981

[25] Hassell TM. Tissues and cells of the periodontium. Periodontol 2000 1993; 3: 9–38. 9673156 10.1111/j.1600-0757.1993.tb00230.x

[26] Andrian E, Grenier D, Rouabhia M. *Porphyromonas gingivalis*-epithelial cell interactions in periodontitis. J Dent Res 2006; 85: 392–403. 16632751 10.1177/154405910608500502

[27] Takashiba S, Naruishi K, Murayama Y. Perspective of cytokine regulation for periodontal treatment: fibroblast biology. J Periodontol 2003; 74: 103–110. 12593604 10.1902/jop.2003.74.1.103

[28] Garlet GP. Destructive and protective roles of cytokines in periodontitis: A reappraisal from host defense and tissue destruction viewpoints. J Dent Res 2010; 89: 1349–1363. 10.1177/0022034510376402 20739705

[29] Irwin CR, Myrillas TT. The role of IL-6 in the pathogenesis of periodontal disease. Oral Dis 1998; 4: 43–47. 9655045 10.1111/j.1601-0825.1998.tb00255.x

[30] Takahashi K, Takashiba S, Nagai A, Takigawa M, Myoukai F, Kurihara H, et al Assessment of interleukin-6 in the pathogenesis of periodontal disease. J Periodontol 1994; 65: 147–153. 8158511 10.1902/jop.1994.65.2.147

[31] Tamura T, Udagawa N, Takahashi N, Miyaura C, Tanaka S, Yamada Y, et al Soluble interleukin-6 receptor triggers osteoclast formation by interleukin-6. PNAS USA 1993; 90: 11924–11928. 8265649 10.1073/pnas.90.24.11924 PMC48097

[32] Ishimi Y, Miyaura C, Jin CH, Akatsu T, Abe E, Nakamura Y, et al IL-6 is produced by osteoblasts and induces bone resorption. J Immunol 1990; 145: 3297–3303. 2121824

[33] Gamonal J, Acevedo A, Bascones A, Jorge O, Silva A. Levels of interleukin-1 beta, -8, and -10 and RANTES in gingival crevicular fluid and cell populations in adult periodontitis patients and the effect of periodontal treatment. J Periodontol 2000; 71: 1535–1545. 11063385 10.1902/jop.2000.71.10.1535

[34] Gamonal J, Acevedo A, Bascones A, Jorge O, Silva A. Characterization of cellular infiltrate, detection of chemokine receptor CCR5 and interleukin-8 and RANTES chemokines in adult periodontitis. J Periodontal Res 2001; 36: 194–203. 11453119 10.1034/j.1600-0765.2001.360309.x

[35] Teng YT, Sodek J, McCulloch CA. Gingival crevicular fluid gelatinase and its relationship to periodontal disease in human subjects. J Periodontal Res 1992; 27: 544–552. 1403585 10.1111/j.1600-0765.1992.tb01830.x

[36] Kubota T, Nomura T, Takahashi T, Hara K. Expression of mRNA for matrix metalloproteinases and tissue inhibitors of metalloproteinases in periodontitis-affected human gingival tissue. Archs Oral Biol 1996; 41: 253–262. 10.1016/0003-9969(95)00126-3 8735011

[37] Yoshida K, Yoshioka M, Okamura H, Moriyama S, Kawazoe K, Grenier D, et al Preventive effect of Daiokanzoto (TJ-84) on 5-fluorouracil-induced human gingival cell death through the inhibition of reactive oxygen species production and NLRP3 inflammasome activation. PloS One 2014; 9: e112689 10.1371/journal.pone.0112689 25389767 PMC4229234

